# Survival outcomes in hormone receptor-negative breast cancer among BRCA carriers versus noncarriers in western Sweden

**DOI:** 10.2340/1651-226X.2025.43109

**Published:** 2025-04-16

**Authors:** Anna-Karin Tzikas, Erik Holmberg, Toshima Z. Parris, Anikó Kovács, Lovisa Lovmar, Per Karlsson

**Affiliations:** a Department of Oncology, Institute of Clinical Sciences, Sahlgrenska Academy, University of Gothenburg, Gothenburg, Sweden; b Department of Oncology, Region Västra Götaland, NU Hospital Group, Uddevalla, Sweden; c Sahlgrenska Center for Cancer Research, Sahlgrenska Academy, University of Gothenburg, Gothenburg, Sweden; d Department of Clinical Pathology, Region Västra Götaland, Sahlgrenska University Hospital, Gothenburg, Sweden; e Department of Clinical Genetics and Genomics, Region Västra Götaland, Sahlgrenska University Hospital, Gothenburg, Sweden; f Department of Oncology, Region Västra Götaland, Sahlgrenska University Hospital, Gothenburg, Sweden

**Keywords:** HR negative, ER negative, TNBC, survival, genetic testing, hereditary breast cancer

## Abstract

**Background and purpose:**

BRCA-related hormone receptor (HR)-negative breast cancers (BC) are reported to have aggressive tumor biology but also exhibit chemosensitivity. However, the impact of *BRCA1/2* pathogenetic variants (PV) on BC outcomes remains unclear. This study compares survival outcomes for HR-negative BC between BRCA carriers and noncarriers.

**Patients/material and methods:**

From 489 female BRCA-carriers prospectively registered in western Sweden (1996–2017), those with primary HR-negative BC who underwent breast surgery until 2019 were included in the BRCA cohort. For each BRCA-carrier, three BRCA-noncarriers with HR-negative BC were matched based on age, time of diagnosis, and follow-up duration. Overall survival (OS) was analyzed using Kaplan‑Meier estimates and Cox proportional hazard ratios after adjustment for stage, chemotherapy, and surgical technique. A sensitivity analysis was performed to investigate the effect of HER2 status on HR-negative BC diagnosed after 2007.

**Results:**

Among the 106 BRCA carriers, 101 (95%) had a *BRCA1* and 5 (5%) a *BRCA2* PV. Most of the BRCA-carriers (89/106, 84%) were diagnosed with BC prior to genetic screening. Surgical techniques were similar between BRCA-carriers (*n* = 106) and noncarriers (*n* = 318). Chemotherapy was more common among BRCA-carriers (87% vs. 72%, *p <* 0.001). No significant difference in OS was found between BRCA-carriers and noncarriers among patients with HR-negative BC (adjusted HR: 0.81 [95% confidence interval [CI]: 0.43–1.53], *p* = 0.51) or considering HER2 status (adjusted HR 0.95 [95% CI: 0.43–2.07], *p* = 0.89).

**Interpretation:**

This study suggests that *BRCA1/2* pathogenic variants do not independently impact survival outcomes in HR-negative BC. However, a moderate association between BRCA status and OS cannot be ruled out.

## Introduction

Despite only accounting for about 15–20% of all breast cancers (BCs), the hormone receptor (HR)-negative subtype, that is, estrogen receptor (ER) and progesterone receptor (PgR) negative, causes a disproportionate number of BC-related deaths the first 5 years after diagnosis [1]. Since its introduction into standard care in 2007, human epidermal growth factor receptor 2 (HER2) testing has facilitated the stratification of HR-negative BCs into two groups: HR-negative, HER2-positive BCs and triple-negative BCs (TNBC; HR negative, HER2 negative). A US cohort study comprised of over 50,000 BCs revealed that 73% of the HR-negative BCs were TNBC [2]. HR-negative BC and TNBC are both associated with a higher likelihood of early metastasis and worse prognosis [3].

Clinically relevant germline pathogenic variants (PV) in *BRCA1* or *BRCA2* are hereditary genetic alterations that increase the risk of developing BC and ovarian cancer (OC). Using a relatively large prospective cohort of BRCA carriers, Kuchenbaecker and colleagues estimated the cumulative BC risk for individuals aged 80 years to be 72% for *BRCA1* and 69% for *BRCA2*, while the cumulative OC risk was 44% for *BRCA1* and 17% for *BRCA2* [4]. Harboring a BRCA PV, especially in *BRCA1*, is associated with an increased risk of developing HR-negative BC, particularly for the TNBC subtype [5, 6]. Since TNBC status alone is currently an indication for BRCA screening, regardless of family history [7], it is not uncommon for patients to be simultaneously diagnosed with both TNBC or HR-negative BC and a germline BRCA PV. The risk of harboring a BRCA PV for those diagnosed with TNBC is estimated to be 10–20% [8, 9], and 60–80% of all *BRCA1*-related BCs are TNBC [10]. Compared with sporadic cases, *BRCA1*-related BCs have been reported to more often be of ductal histological type, grade 3, and contain severe lymphocyte infiltration [11]. Nevertheless, BRCA-related BCs have also been shown to be chemosensitive [12].

*BRCA1* was identified in 1994 and *BRCA2* in 1995–1996 [13–15]; at the same time, the oncogenetic clinic was established in Gothenburg, Sweden. In 2001, Einbeigi et al. described a common founder *BRCA1* mutation in western Sweden [16]. The same year, a regional follow-up register for BRCA carriers (BRCA register) was established in western Sweden to ensure individuals were offered adequate risk-reducing surgery, follow-up controls, and the evaluation of long-term outcomes. Using the BRCA register, Öfverholm *et al.* previously reported an increase in overall mortality among BRCA carriers despite risk-reducing surgery [17]. However, it is still not clear whether BRCA PVs, particularly for HR-negative BC, have an adverse impact on prognosis [18].

BC patients harboring PVs in *BRCA1* or *BRCA2* (BRCA carriers) often inquire whether these PVs will affect their prognosis. In the present study, we used data from the BRCA register to compare overall survival for BRCA carriers and noncarriers diagnosed with primary HR-negative BC (1987-2019).

## Patients/material and methods

### Study design and setting

This observational study was conducted in the western healthcare region of Sweden, which includes Västra Götalandsregionen (VGR) and northern Halland, and comprises one university hospital and five county hospitals. The Western healthcare region is one of the six healthcare regions in Sweden, serving approximately 2 million inhabitants – about one-fifth of the total population of the country [19, 20]. The socioeconomic situation in the Western healthcare region is similar to that of Sweden overall. The study complies with the STROBE guidelines for reporting [21]. We used the regional BRCA follow-up register, henceforth referred to as the ‘BRCA register’, to identify a prospectively registered HR-negative BC cohort of BRCA carriers. The BRCA register included women who had tested positive for a BRCA PV between 1996 and 2017 at the regional oncogenetic clinic in the western healthcare region of Sweden. Managed by the regional oncogenetic clinic, the register was set up to ensure that individuals were offered adequate risk-reducing surgery, follow-up controls, and long-term outcome assessments. The quality of the register was assessed in 2017 [17]. Patients were referred to the regional oncogenetic clinic for BRCA screening according to the previous and current national healthcare programs [22], which are generally similar to international guidelines [7].

For the control group, patients with HR-negative BC were selected from a regional BC registry (VGR, Halland) for 1987–2007 and the Swedish National Breast Cancer Quality Register (NKBC) for 2007–2019 (due to reorganization of the Swedish Breast Cancer Registers in 2007). Both the regional BC registry and NKBC will henceforth be collectively referred to as the ‘breast cancer registry’. HR-negative BC was defined as both ER- and PR-negative (≤10% ER and PR expression), and HER2-positive BC was defined as HercepTest 3+ or 2+ and SISH/FISH amplified; TNBC was defined as ER negative, PR negative, and HER2 negative (HercepTest 0 or 1+ or 2+ and SISH/FISH not amplified), according to national Swedish guidelines [22]. After 2007, the completeness of the breast cancer registry for ER, PR, and HER2 status exceeded 96% [23]. Information about primary tumor characteristics, TNM, and treatment (surgery, chemotherapy, and radiotherapy) was collected from the breast cancer registry and follow-up data retrieved January 31, 2021.

### Study population

Inclusion criteria for both the BRCA cohort and control group were a diagnosis of primary T1-T3 HR-negative BC between 1987 and 2019 in western Sweden and who had undergone breast surgery to ensure that only staged patients with histopathologically verified HR-negative BC were included. Individuals with T4 tumors or missing T status were excluded. Patients diagnosed with distant metastases within 3 months of diagnosis and patients with a history of other cancers (other than squamous cell carcinoma of the skin) within a 10-year period prior to the first HR-negative breast cancer diagnosis were also excluded.

### BRCA cohort

Women who tested positive for disease-causing variants in BRCA genes (*BRCA1* or *2*) between 1996 and 2017 were screened for eligibility using the BRCA register. The BRCA cohort included women harboring a BRCA PV and diagnosed with HR-negative BC between 1987 and 2019 in western Sweden (before or after BRCA testing). The BRCA register was then used to collect information on the date of DNA analysis, the PV gene (*BRCA1* or *2*), date of risk-reducing mastectomy, date of risk-reducing oophorectomy, date and type of any cancer diagnosis after registration, time between DNA testing and BC diagnoses, age at BC diagnosis, date of death, and cause of death.

### Control group

To identify patients for the control group, HR-negative BCs diagnosed in western Sweden between 1987 and 2019 were selected from the breast cancer registry and screened for eligibility. Patients were assumed to be noncarriers if they were not found in the BRCA register. Known BRCA carriers identified using the BRCA register were excluded from the control group.

### Patient matching

Three matched controls, assumed to be BRCA noncarriers, were identified per corresponding BRCA carrier based on age at diagnosis, diagnosis period (*<2008 or ≥2008* when HER2 status was complete), followed up and being alive at least during the period between the BC diagnosis and BRCA testing. This was performed to avoid survival bias in the BRCA carriers when BRCA screening was performed a long time after the BC diagnosis. The age matching criteria were:

- ≤ 35 years => age of the control ± 5 years
- > 35 – ≤45 years => age of the control ± 3 years
- > 45 years => age of the control ± 1 years

No patients in the control group were reused, and patients were randomly selected from those who met the matching criteria.

### Sensitivity analysis

When adequately treated, HER2-positive, HR-negative patients have a better prognosis than TNBCs. Therefore, a sensitivity analysis including patients diagnosed after 2007 (when HER2 status was available and included in the register) was performed to examine whether HER2 status would affect overall survival. We selected BRCA carriers from the BRCA cohort with a BRCA PV and HR-negative BCs diagnosed between 2007 and 2019. For each BRCA carrier, we selected three new patients with HR-negative BC (diagnosed 2007–2019) from the breast cancer registry for the control group. Patient matching was performed based on age at diagnosis, HER2 status, and followed up and alive at least during the period between the BC diagnosis and BRCA testing for the corresponding BRCA carrier.

### Statistical analyses

In the present study, the primary outcome was overall survival (OS). Follow-up time was measured from HR-negative breast cancer diagnosis until death occurred or censored at last follow-up. The data were analyzed up to 10 years after BC diagnosis. OS was analyzed using Kaplan‑Meier estimates and log-rank test. Cox proportional hazards models were used to estimate hazard ratio (HR), adjusted for tumor stage, adjuvant/neoadjuvant chemotherapy, and surgical technique (mastectomy/partial mastectomy). The Schoenfeld residuals was used to check the proportional hazards assumption. All covariates in the models fulfilled the assumption. *P*-values <0.05 for a two-sided test were considered statistically significant. Stata statistical software version 18.1 for Mac (StataCorp. 2024, College Station, TX: StataCorp LLC) was used for all statistical analyses.

## Results

### Baseline patient characteristics

**Figure 1:**
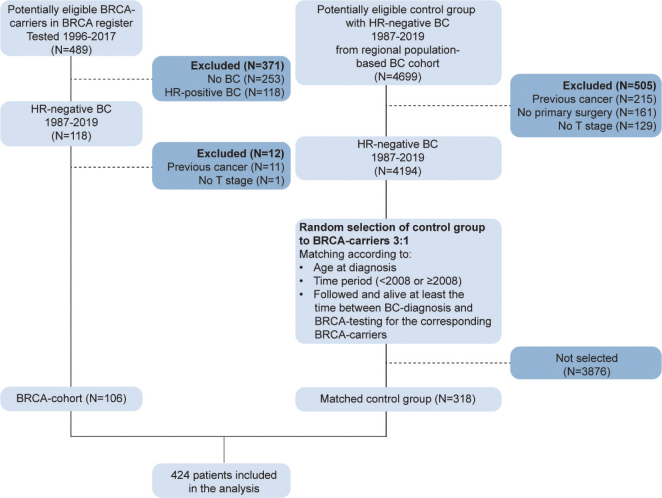
Flowchart describing the inclusion of the 424 patients in the BRCA cohort and matched control group with HR-negative breast cancer.

Using the BRCA register, 489 female BRCA carriers were registered between 1997 and 2017, of which 236 had developed breast cancer during 1987–2019, and 118 of 236 (50%) of the cases were classified as HR-negative BC (Figure 1). In total, 106 of 118 patients with HR-negative BC fulfilling the inclusion criteria were selected for the BRCA cohort. For the control group, 318 matched patients were identified from 4194 patients in the regional BC cohort selected from the breast cancer registry. The study population therefore consisted of 424 patients with HR-negative BC, including 106 in the BRCA cohort and 318 in the control group (referred to as BRCA noncarriers).

**Figure 2:**
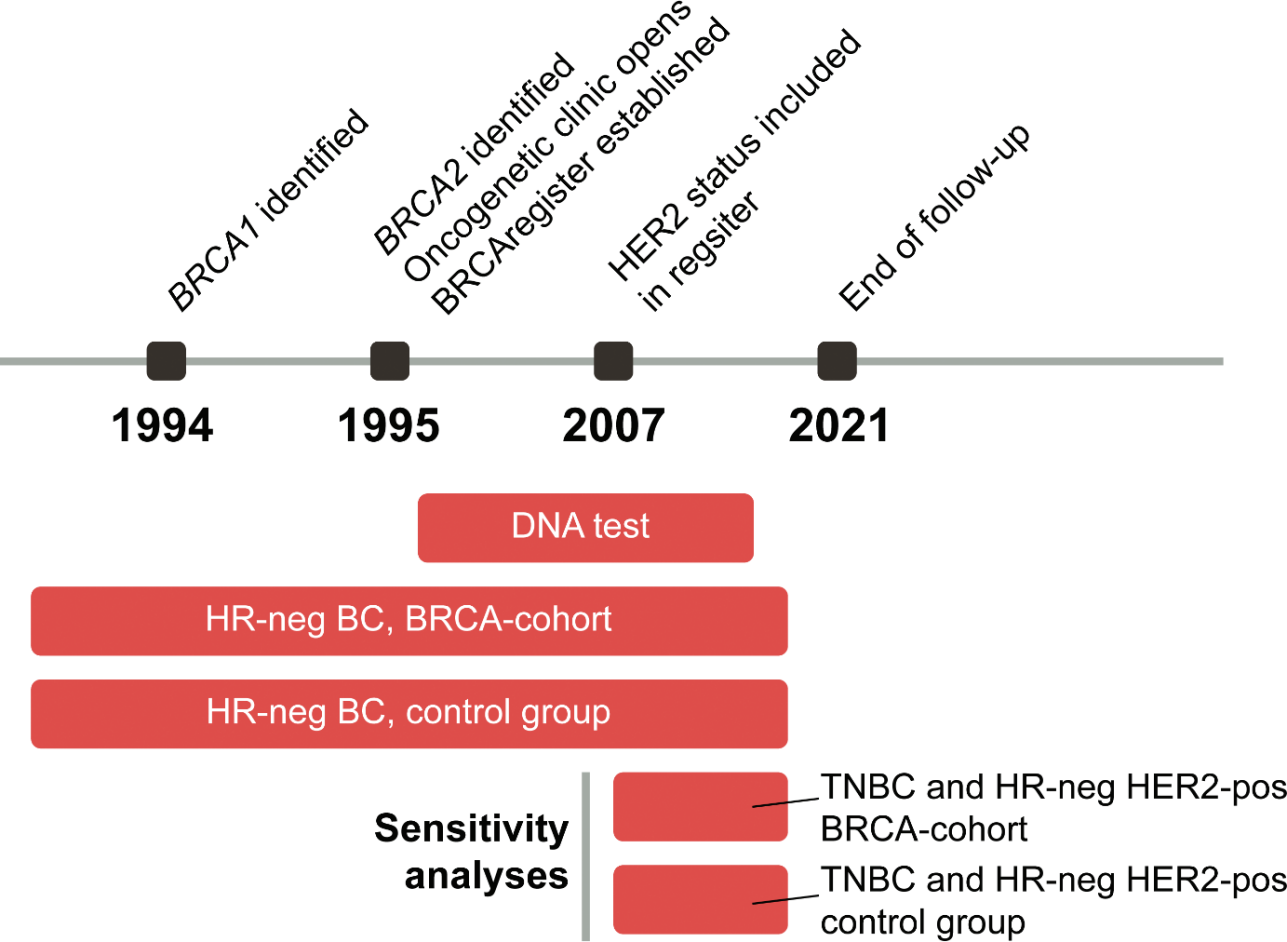
Timeline showing years of identification of *BRCA1 and 2*, start of oncogenetic clinic, BRCA register, and timing of DNA tests and BC diagnoses of the current study as well as for the additional sensitivity analysis. End of follow-up was January 31, 2021.

The median age at diagnosis was 43 years (IQR, 36–51 years), with most patients having T2 tumors (Table 1). Age and initial surgical techniques were similar in the BRCA cohort and control group. However, adjuvant/neoadjuvant chemotherapy was used more frequently in the BRCA cohort (87% vs. 72%, *p* < 0.001), while lymph node involvement (24% vs. 38%, *p* = 0.015) and radiation therapy (50% vs. 64%, *p* = 0.025) were more common in the control group. In the BRCA cohort, 101 patients (95%) had a *BRCA1* PV and 5 (5%) had a *BRCA2* PV. Furthermore, 20 of 106 BRCA carriers had bilateral breast cancer, of which eight were synchronous and three patients also had a subsequent ovarian cancer. After primary breast cancer surgery, 17 of 106 had risk-reducing mastectomy and 30 of 106 had bilateral salpingo-oophorectomy. Figure 2 outlines the timing of *BRCA1* and *2* identification, start of oncogenetic clinic, BRCA register, DNA tests, and BC diagnoses of the current study as well as for the additional sensitivity analysis.

**Table 1:** Population characteristics.

	Total	BRCA cohort	Control group	*p*
Age at diagnosis, mean (SD)	43.6 (9.1)	43.2 (9.4)	43.7 (9.0)	0.63
Age at diagnosis, median (IQR)	42.9 (36.3–50.5)	42.4 (35.2–50.3)	43.0 (36.4–50.5)	0.61
Age group (years)				0.83
20–34	86 (20.3%)	26 (24.5%)	60 (18.9%)	
35–39	86 (20.3%)	19 (17.9%)	67 (21.1%)	
40–44	76 (17.9%)	18 (17.0%)	58 (18.2%)	
45–49	60 (14.2%)	15 (14.2%)	45 (14.2%)	
50–54	64 (15.1%)	14 (13.2%)	50 (15.7%)	
> 54	52 (12.3%)	14 (13.2%)	38 (11.9%)	
T (cm)				0.46
0–2	165 (38.9%)	45 (42.5%)	120 (37.7%)	
> 2–5	215 (50.7%)	53 (50.0%)	162 (50.9%)	
> 5	44 (10.4%)	8 ( 7.5%)	36 (11.3%)	
*N*				0.015
N0	268 (63.2%)	75 (70.8%)	193 (60.7%)	
N+	145 (34.2%)	25 (23.6%)	120 (37.7%)	
Missing	11 ( 2.6%)	6 ( 5.7%)	5 ( 1.6%)	
Initial breast surgery Partial mastectomy	205 (48.3%)	49 (46.2%)	156 (49.1%)	0.61
Total mastectomy	219 (51.7%)	57 (53.8%)	162 (50.9%)	
Radiation therapy				0.025
No	139 (32.8%)	43 (40.6%)	96 (30.2%)	
Yes	255 (60.1%)	53 (50.0%)	202 (63.5%)	
Missing	30 ( 7.1%)	10 ( 9.4%)	20 ( 6.3%)	
Chemotherapy				< 0.001
No	78 (18.4%)	8 ( 7.5%)	70 (22.0%)	
Yes	322 (75.9%)	92 (86.8%)	230 (72.3%)	
Missing	24 ( 5.7%)	6 ( 5.7%)	18 ( 5.7%)	

**Figure 3:**
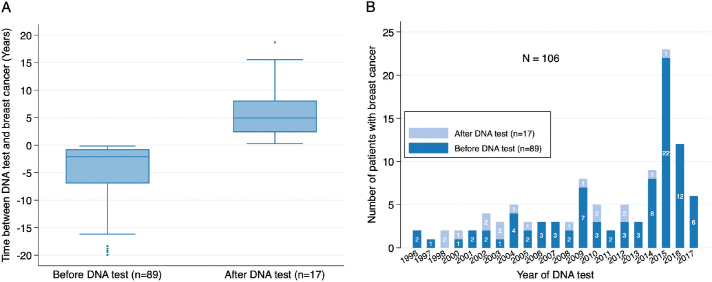
(A) Boxplots showing the time between DNA test and breast cancer diagnosis. (B) Stacked bar plots illustrating the distribution of DNA test and time for breast cancer.

A majority of the individuals in the BRCA cohort (89 of 106) had a history of HR-negative BC before BRCA testing (Figure 3A), with an increase in the detection of BRCA-related HR-negative BCs over time (Figure 3B). The median time from BC to BRCA testing was 2.1 years (range, 0.2–19.9 years). Seventeen of the 106 BRCA carriers (16%) had a known BRCA PV before their BC diagnosis. The median time from BRCA testing to BC diagnosis was 4.9 years (range, 0.3–18.7 years) in this group. Among the 17 BRCA carriers who had a known BRCA PV before their BC diagnoses, none had performed risk-reducing breast surgery before their BC diagnosis. Most of them (16 of 17) received a mastectomy at the time of BC diagnosis, and T1 tumors were more prevalent in this group compared to patients that were BRCA tested after BC (76% vs. 36%, *p* = 0.012). No difference was found regarding lymph node involvement.

### Follow-up and survival

**Figure 4:**
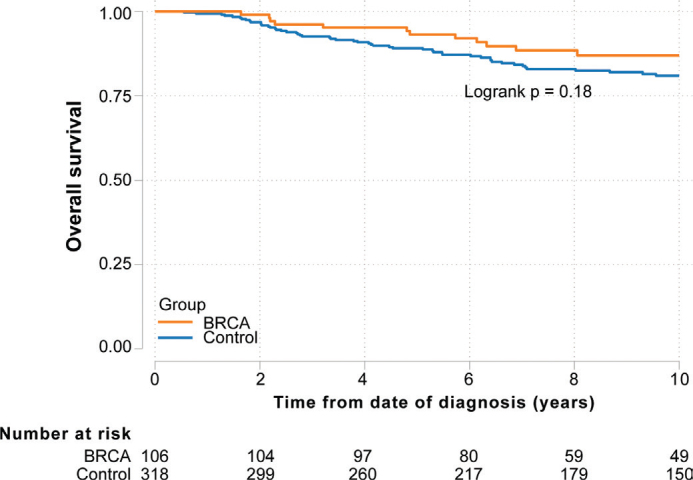
Kaplan‑Meier plot showing overall survival according to BRCA status.

The median follow-up time after BC was similar between the BRCA cohort (8.9 years [range, 1.6–27.3 years]) and control group (9.5 years [range, 0.6–30.1 years]). Furthermore, no significant difference in OS was found between the BRCA cohort and control group (unadjusted: HR 0.65 [95% confidence interval [CI]: 0.35–1.22], *p* = 0.18, Table 2, Figure 4; adjusted [stage, chemotherapy, and surgical technique]: HR 0.81 [95% CI: 0.43–1.53], *p* = 0.51). In the sensitivity analysis, 61 of the 106 BRCA-related HR-negative BCs who were diagnosed between 2007 and 2019 were selected (Supplemental Figure 1 and Supplemental Table 1). Ninety-five percent of the BRCA-related BCs were TNBC and 5% HR negative, HER2 positive. No significant difference in OS was found between BRCA carriers and noncarriers in the sensitivity analysis (unadjusted: HR 0.72 [95% CI: 0.34–1.50], *p* = 0.38; adjusted: HR 0.95 [95% CI: 0.43–2.07], *p* = 0.89, Supplementary Table 2, Supplementary Figure 2). Thus, HER2 status had no significant impact on the HRs of OS in carriers versus noncarriers with HR-negative breast cancer.

**Table 2:** Overall survival, cox proportional hazard regression.

Variable	Number of deaths/patients (%) 64/424 (15%)	Univariable		Multivariable	
		HR (95% CI)	*P*	HR (95% CI)	*P*
**Group**					
Control group	52/318 (16%)	Reference		Reference	
BRCA cohort	12/106 (11%)	0.65 (0.35–1.22)	0.177	0.81 (0.43–1.53)	0.512
**Age,** per 10 years					
20–34	18/86 (12%)	Reference		Reference	
35–39	11/86 (13%)	1.19 (0.50–2.79)	0.696	1.32 (0.54–3.19)	0.541
40–44	16/76 (21%)	2.09 (0.95–4.61)	0.068	2.54 (1.10–5.05)	0.029
45–49	11/60 (18%)	1.72 (0.73–4.05)	0.216	2.45 (1.01–5.97)	0.049
50–54	9/64 (14%)	1.29 (0.52–3.18)	0.578	1.69 (0.66–4.29)	0.272
>54	7/52 (13%)	1.21 (0.46–3.18)	0.698	1.73 (0.64–4.71)	0.281
**T, cm**					
0–2	21/165 (13%)	Reference		Reference	
>2–5	31/215 (14%)	1.18 (0.68–2.04)	0.568	1.02 (0.58–1.81)	0.943
>5	12/44 (27%)	2.43 (1.20–4.94)	0.014	2.16 (1.03–4.55)	0.043
***N***					
N0	26/268 (10%)	Reference		Reference	
N+	36/145) (25%)	2.85 (1.72–4.72)	<0.001	2.76 (1.64–4.63)	<0.001
Missing	2/11 (18%)				
**Chemotherapy**					
No	9/78 (12%)	Reference			
Yes	53/322 (16%)	1.35 (0.66–2.73)	0.409		
**Surgery technique**					
Partial mastectomy	24/205 (12%)	Reference			
Total mastectomy	40/219 (18%)	1.61 (0.97–2.67)	0.066		

The proportional hazards assumption was tested and found to be valid.

HR: hazard ratio; CI: confidence interval.

## Discussion and conclusion

In the present study, we aimed to examine the association between BRCA status (carriers vs. noncarriers) and survival after a HR-negative BC diagnosis. We found no significant difference in overall survival among BRCA carriers compared to noncarriers. A sensitivity analysis performed to address HER2 status for patients diagnosed after 2007 (when HER2 testing was included in the Swedish breast cancer registry) support these findings. All HRs of OS between carriers and noncarriers were below one arguing against an adverse effect of BRCA on survival in HR-negative breast cancer. However, the confidence intervals were wide so also an adverse effect of BRCA positivity on HR-negative BC prognosis cannot be ruled out.

BRCA-related HR-negative BCs are aggressive but also more sensitive to chemotherapy [5]. Yet, the effect of BRCA status on overall outcome after BC remains unclear. Previous studies have shown conflicting results with similar, better, or worse survival for BRCA carriers. A meta-analysis could not support lower BC survival for BRCA carriers [18]. Few population-based studies exist that compare the effect of BRCA status on survival [24, 25]. To our knowledge, the only prospective trial to address this issue is the POSH study cohort study comprised of young onset BC, where BC outcome in patients under 40 years of age was compared in BRCA carriers versus noncarriers [26]. No difference in survival was found between BRCA carriers and noncarriers at 2, 5, and 10 years. Outcomes in BRCA carriers with TNBC were found to be better after 2 years, but no difference was found after 5 and 10 years. The observed better survival after 2 years may be attributed to the chemosensitivity of BRCA-related TNBCs.

A strength of the present study is the long follow-up time. Since HR-negative BCs that relapse, typically do so at an early stage within the first 5 years [1], our study includes sufficient follow-up time with a median of 9 years. Additionally, another strength of this study is the completeness of the registry data used to collect information for the control group. One limitation of the current study is the small sample size of the BRCA cohort. Referral criteria for BRCA screening at the oncogenetic clinic has changed over time and were not as wide in the 1990s and early 2000s as current testing criteria. For example, TNBC irrespective of age was not included in the criteria for BRCA screening in Swedish national guidelines until 2017. Therefore, it is possible that the BRCA cohort used in the present study during this period may be incomplete, and that the control group might also include some BRCA carriers. However, the hidden number of BRCA carriers in the control group is expected to be low since the risk of harboring a BRCA PV when diagnosed with TNBC is estimated to be 10–20% [8, 9], and a majority of these most likely are found in the BRCA cohort. It is therefore unlikely that the hidden number of BRCA carriers would affect our results. The wider criteria for BRCA screening seen over the years, including more common use of treatment-focused BRCA screening, are probably the explanation for the increased detection of BRCA-related HR-negative BCs seen over time in this study.

A second limitation of this study is that the analysis is retrospective. Additionally, one could suspect that overall survival in our analysis is slightly better than in the whole group of HR-negative breast cancer since patients in the BRCA cohort had to survive at least until DNA-testing, and the control group was matched according to this. However, this was the same in both groups and therefore could not affect the comparison between BRCA cohort and control group.

Our primary aim was to examine survival after HR-negative breast cancer in BRCA carriers compared with BRCA noncarriers. Because HER2 status was not available in the registers until 2007, we could only adjust for HER2 status in the later period. Before the introduction of HER2-targeted therapy, HER2-positive breast cancer was an aggressive disease with an adverse prognosis. Nowadays, HER2-positive HR-negative BCs have a better prognosis than TNBCs when adequately treated with chemotherapy and HER2-targeted therapy [27], which could influence the results in the latter period of our study. A sensitivity analysis to investigate the influence of HER2 status on our results was performed, including patients diagnosed with BC after 2007 when HER2 status was available. Yet again, no change in OS could be demonstrated. In line with previous results, the sensitivity analysis showed that 58 of 61 (95%) BRCA-related HR-negative BCs were also TNBC. Previous studies have shown that HR-negative tumors in BRCA carriers are predominantly TNBC [6]. In the Swedish SWEA study, 95 and 88% of the HR-negative BC cases among *BRCA1* and *BRCA2* carriers were TNBC, respectively [20]. An analysis of survival differences in the latter group with matched controls produced similar results, indicating that BRCA-related HR-negative BC could be used as a proxy for BRCA-related TNBCs. Although a higher proportion of the HR-negative BCs among BRCA carriers in our study may be TNBC than in the control group, this did not seem to have an adverse effect on prognosis.

One could predict that the BRCA carriers in this study would be diagnosed with smaller tumors due to more intense breast cancer screening in this group compared with controls. This was correct for the few patients (*n* = 17) who had a known BRCA status before their BC diagnosis, but this did not affect the results since the majority did not know their BRCA status before their initial BC diagnosis.

HR-negative BC remains an aggressive disease with adverse outcomes, but many new treatments have been developed and introduced in recent years. For example, neoadjuvant chemotherapy including Carboplatin is now a standard treatment for most TNBCs with the addition of Pembrolizumab for high-risk TNBCs. The neoadjuvant approach gives us the possibility to adjust treatment according to response and add extra treatment if residual disease is present at the time of surgery. During neoadjuvant treatment, there is also time for BRCA screening. Due to increased awareness and availability of BRCA screening today, more patients with HR-negative BC now have a known BRCA status at the time of initial breast cancer surgery and can be offered suitable surgery directly and be spared additional subsequent risk-reducing mastectomy if found to be a BRCA carrier.

Treatment with Pembrolizumab and Atezolizumab has also shown improved outcomes in the metastatic setting for PD-1/PD-L1-positive patients, while PARP inhibitors have also shown better outcomes for BRCA-related BC patients both in the adjuvant and palliative settings [28–33]. The antibody conjugate sacituzumab govitecan was approved for metastatic TNBC, showing improved survival versus physicians’ choice [34]. At this point in time, one could argue that BRCA carriers today are offered targeted therapies like PARP inhibitors and that this would improve their prognoses compared to noncarriers, but these therapies were not available at the time of the analysis and therefore could not affect our results.

A previous regional study has shown an increased risk of overall mortality among BRCA carriers compared with the general population, even after risk-reducing surgery [17]. We could not demonstrate worse survival after HR-negative BC among BRCA carriers compared with noncarriers. However, the number of individuals is small and confidence intervals wide; therefore, a difference between the groups cannot be excluded. Further research and prospective data are needed to investigate whether BRCA status is a prognostic marker among HR-negative BC patients, especially today when more targeted therapy for BRCA-related BC is available.

## Supplementary Material

Survival outcomes in hormone receptor-negative breast cancer among BRCA carriers versus noncarriers in western Sweden

## Data Availability

De-identified data available upon request.
