# Supplementary material for: Survival outcomes in hormone receptor-negative breast cancer among BRCA carriers versus noncarriers in western Sweden
Source: Acta Oncol. 2025 Apr 16;64:43109. doi: 10.2340/1651-226X.2025.43109 (PMC12016662; doi:10.2340/1651-226X.2025.43109)

Supplementary material has been published as submitted. It has not been copyedited, or typeset by Acta Oncologica

Supplement figure 1. Patient selection for the sensitivity analysis for BRCA-carriers and noncarriers with HR-negative breast cancer 2007-2019 and known HER2 status.

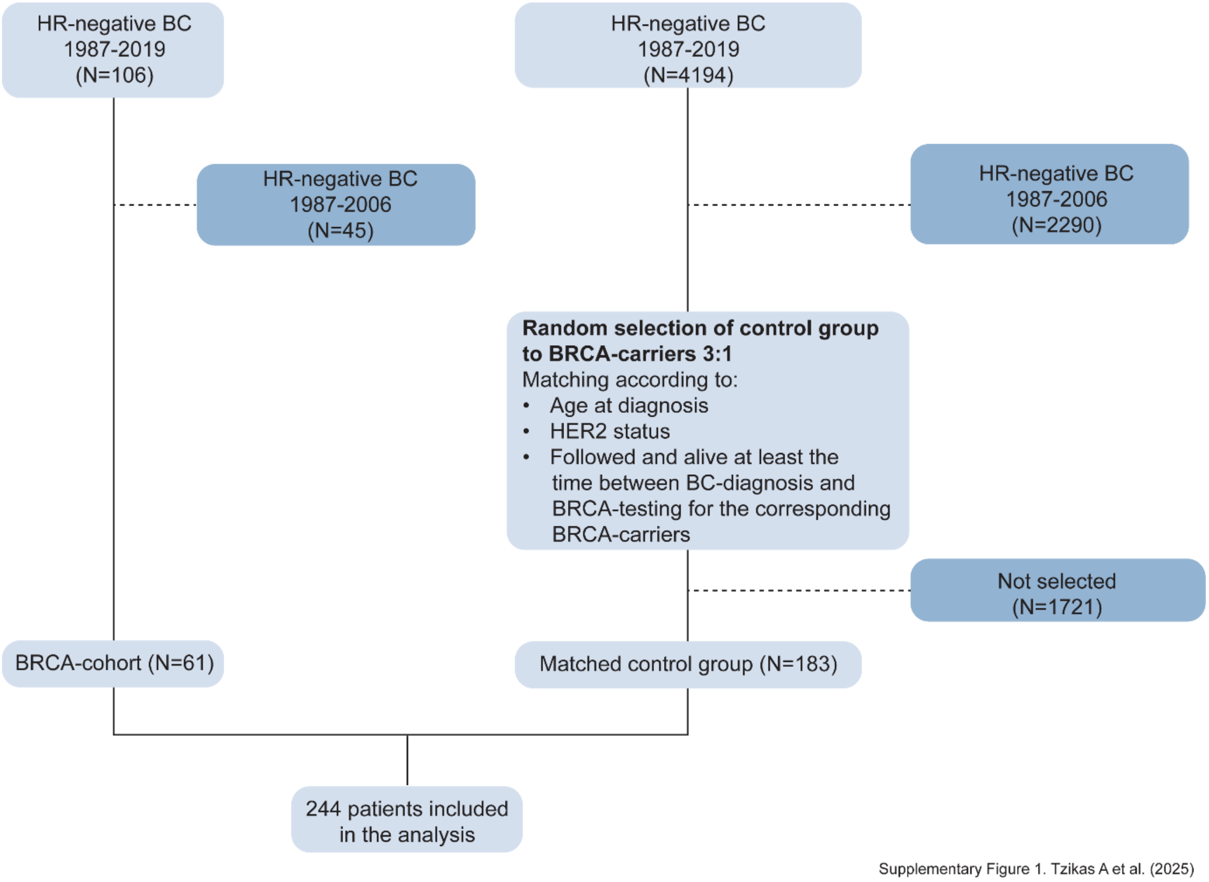

Supplement table 1. Population characteristics, sensitivity analysis BC 2007-2019

	**Ctrl group N=183**	**BRCA N=61**	**Total N=244**	**p-value**
Age at diagnosis, mean (SD)	44.5 (9.0)	44.3 (9.1)	44.5 (9.0)	0.85
Age at diagnosis, medianb (IQR)	44.1 (36.5-51.3)	43.8 (37.3-51.4)	44.1 (36.6-51.3)	0.83
Age group				0.98
20-34	30 (16.4%)	12 (19.7%)	42 (17.2%)	
35-39	35 (19.1%)	11 (18.0%)	46 (18.9%)	
40-44	29 (15.8%)	11 (18.0%)	40 (16.4%)	
45-49	32 (17.5%)	10 (16.4%)	42 (17.2%)	
50-54	36 (19.7%)	10 (16.4%)	46 (18.9%)	
>54	21 (11.5%)	7 (11.5%)	28 (11.5%)	
T(cm)				0.62
0-2	71 (38.8%)	28 (45.9%)	99 (40.6%)	
>2-5	99 (54.1%)	29 (47.5%)	128 (52.5%)	
>5	13 (7.1%)	4 (6.6%)	17 (7.0%)	
N				0.016
N0	110 (60.1%)	45 (73.8%)	155 (63.5%)	
N+	70 (38.3%)	12 (19.7%)	82 (33.6%)	
Missing	3 (1.6%)	4 (6.6%)	7 (2.9%)	
HER2				1.00
Positive	9 (4.9%)	3 (4.9%)	12 (4.9%)	
Negative	174 (95.1%)	58 (95.1%)	232 (95.1%)	
Surgery technique				0.018
Partial mastectomy	102 (55.7%)	23 (37.7%)	125 (51.2%)	
Total mastectomy	81 (44.3%)	38 (62.3%)	119 (48.8%)	
RT				<0.001
No	40 (21.9%)	31 (50.8%)	71 (29.1%)	
Yes	126 (68.9%)	26 (42.6%)	152 (62.3%)	
Missing	17 (9.3%)	4 (6.6%)	21 (8.6%)	
Chemotherapy				0.096
No	23 (12.6%)	3 (4.9%)	26 (10.7%)	
Yes	143 (78.1%)	54 (88.5%)	197 (80.7%)	
Missing	17 (9.3%)	4 (6.6%)	21 (8.6%)	

Supplement table 2.

Cox proportional hazard regression for overall survival, 10-year follow-up, sensitivity analysis BC 2007-2019

Variable	Number of deaths/patients (%) 43/244 (18%)	Univariable		Multivariable	
		HR (95%CI)	P	HR (95%CI)	P
**Group**					
Control group	34/183 (34%)	Reference		Reference	
BRCA cohort	9/61 (15%)	0.72 (0.34-1.50)	0.378	0.95 (0.43-2.07)	0.891
**Age,** per 10 years					
20-34	7/42 (17%)	Reference			
35-39	8/46 (17%)	0.98 (0.36-2.70)	0.969		
40-44	7/40 (18%)	1.01 (0.35-2.88)	0.985		
45-49	10/42 (24%)	1.35 (0.51-3.55)	0.541		
50-54	8/46 (17%)	0.98 (0.35-2.70)	0.966		
>54	3/28 (11%)	0.63 (0.16-2.43)	0.501		
**T, cm**					
0-2	13/99 (13%)	Reference			
>2-5	23/128 (18%)	1.42 (0.72-2.80)	0.314		
>5	7/17 (41%)	4.15 (1.65-10.4)	0.002		
**N**					
N0	18/155 (12%)	Reference		Reference	
N+	24/82 (29%)	2.85 (1.55-5.26)	0.001	2.52 (1.30-4.88)	<0.001
Missing	1/7 (14%)				
**Chemotherapy**					
No	9/26 (35%)	Reference		Reference	
Yes	30/197 (15%)	0.26 (0.12-0.55)	<0.001	0.28 (0.13-0.61)	0.001
Missing	4/21 (19%)				
**Surgery technique**					
Partial mastectomy	13/125 (10%)	Reference		Reference	
Total mastectomy	30/119 (25%)	2.51 (1.31-4.82)	0.006	2.03 (1.01-4.08)	0.047

The proportional hazards assumption was tested and found to be valid.

HR = Hazard Ratio, CI = Confidence Interval.

Supplement figure 2. Kaplan-Meier plot showing overall survival according to BRCA-status, sensitivity analysis BC 2007-2019

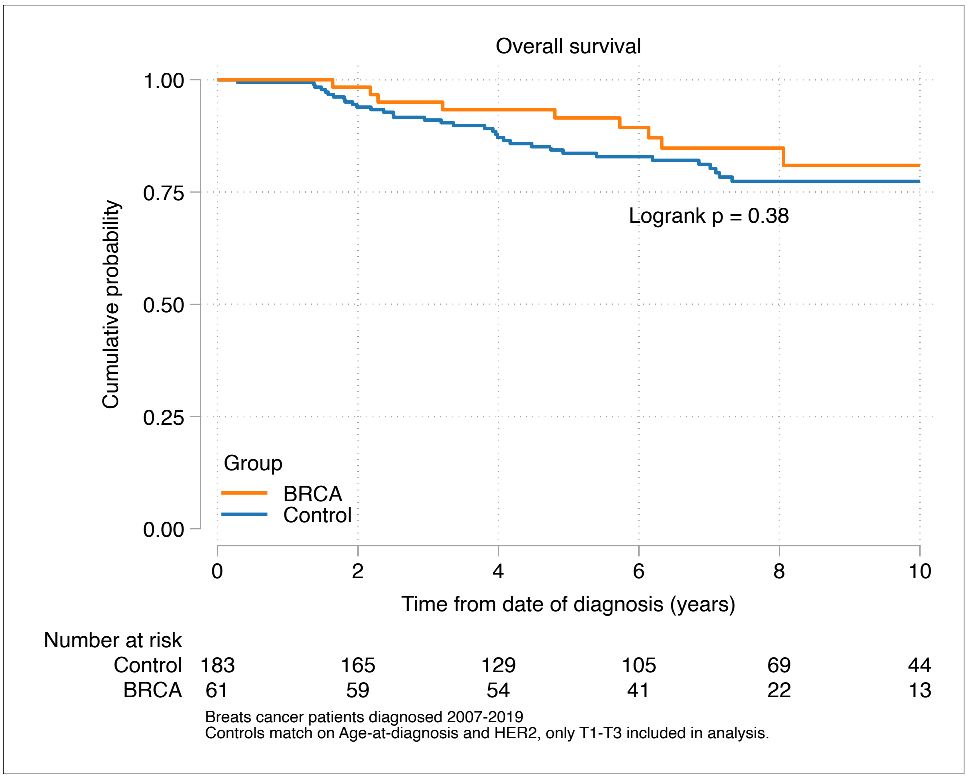

Supplement: Survival outcomes in hormone receptor-negative breast cancer among BRCA carriers versus noncarriers in western Sweden [file AO-64-43109-s1.pdf]
